# Impact of Final Irrigation Protocol on the Push-Out Bond Strength of Two Types of Endodontic Sealers

**DOI:** 10.3390/ma16051761

**Published:** 2023-02-21

**Authors:** Germain Sfeir, Frédéric Bukiet, Wajih Hage, Roula El Hachem, Carla Zogheib

**Affiliations:** 1 Department of Endodontics, Faculty of Dental Medicine, Saint Joseph University of Beirut, Beirut 17-5208, Lebanon; 2 Assistance Publique des Hôpitaux de Marseille, 13005 France; Aix Marseille Univ, CNRS, ISM, Inst. Movement Sci, 13288 Marseille, France

**Keywords:** calcium silicate-based sealers, failure, HEDP, irrigation, push-out bond strength, single cone

## Abstract

*Aim:* The aim of this study was to assess the impact of the final irrigation protocol on the push-out bond strength of calcium silicate-based sealers when compared to an epoxy resin-based sealer. *Materials and Methods:* Eighty-four single-rooted mandibular human premolars were shaped using the R25^®^ instrument (Reciproc, VDW, Munich, Germany) and then divided into three subgroups of 28 roots each depending on the final irrigation protocol: EDTA (ethylene diamine tetra acetic acid) and NaOCl activation, Dual Rinse^®^ HEDP (1-hydroxyethane 1,1-diphosphonate) activation or sodium hypochlorite (NaOCl) activation. Then, each subgroup was divided into two groups (14 each) according to the sealer used (AH Plus Jet^®^ or Total Fill BC Sealer^®^) for single-cone obturation. Dislodgement resistance using a universal testing machine, samples’ push-out bond strength and failure mode under magnification were determined. *Results:* EDTA/Total Fill BC Sealer^®^ showed significantly greater values of push-out bond strength compared with HEDP/Total Fill BC Sealer^®^ and NaOCl/AH Plus Jet^®^, with no statistical difference with EDTA/AH Plus Jet^®^, HEDP/AH Plus Jet^®^ and NaOCl/Total Fill BC Sealer^®^, whereas HEDP/Total Fill BC Sealer^®^ showed significantly lower values of push-out bond strength. The apical third demonstrated higher means of push-out bond strength compared with middle and apical thirds. The most common failure mode was cohesive but showed no statistical difference compared to other types. *Conclusions*: Irrigation solution and final irrigation protocol affect the adhesion of calcium silicate-based sealers.

## 1. Introduction

Endodontic treatment outcome is directly correlated with the quality of the root canal disinfection and sealing [1]. It is well accepted that shaping and irrigation play a key role in minimizing the microbial load and ensuring the chemical debridement of the endodontic system [2]. It has also been demonstrated that smear layer removal can optimize adhesion of the endodontic sealers to dentin walls [3]. Moreover, an enhanced obturation quality through an optimum bond between gutta-percha/sealer and dentin prevents coronal and apical micro leakage [4].

Lately, several hydraulic calcium silicate-based sealer (CSBS) formulations have been introduced. They have gained popularity in the last decade considering their specific behavior, especially their improved biological properties and their association with the updated single-cone technique [5,6,7]. CSBSs have the ability to create a chemical interaction with dentin walls, showing the formation of apatite precursors or hydroxyapatite tag-like structures [8,9]. This layer is known as the “mineral infiltration zone”, where the alkaline caustic effect of the CSBS hydration products degrades the collagenous component of the interfacial dentin, leading to an increased ion diffusion [10]. However, their adhesion to gutta-percha remains unsatisfactory even when using specific pre-impregnated gutta-percha cones [11] which were initially claimed to enhance this connection [12].

The push-out bond strength (POBS) is widely used to assess the dislodgment resistance of endodontic sealers, thus evaluating their adhesion to dentinal walls and gutta-percha [13]. CSBS hydrophilic nature and gutta-percha’s hydrophobic surface can influence POBS, explaining why “cohesive” failures are most often observed in previous studies [14,15,16]. The bond strength of CSBS can also be affected by their chemical composition, the irrigation protocol [15,17] and the filling technique [10,15,18]. This might explain the results of previous studies showing that smear layer removal using EDTA may reduce the POBS of CSBS [19,20].

Recently, Dual Rinse^®^ HEDP (Medcem GmbH, Weinfelden, Switzerland), also known as etidronic acid (1-hydroxyethylidene-1,1-diphosphonic acid), has been introduced. It consists of a “soft” chelator that can be used in direct combination with NaOCl to form an all-in-one disinfecting, deproteinizing and chelating irrigant [21]. HEDP is claimed to be less aggressive on dentin than EDTA because of its reduced chelating capacity [22]. However, rather than removing a smear layer that has already been formed, HEDP and NaOCl together prevent smear layer formation during instrumentation, leading to the concept of “continuous chelation” [23] with better adhesion of obturation materials to dentin walls [24].

Consequently, chelating and deproteinizing the root canal walls during preparation/irrigation may enhance disinfection of the root canal space and the POBS of endodontic sealers [25].

To date, the impact of HEDP mixed with NaOCl on the POBS of CSBS has been investigated in only one study showing no detrimental effect on the POBS of three calcium silicate-based materials when used in the treatment of simulated dentin slices perforations [26]. Overall, there is still a knowledge gap regarding the relationship between final irrigation protocol, root dentin conditioning and the adhesion of CSBS.

Therefore, the main goal of this study was to evaluate the impact of final irrigation protocol/dentinal surface preparation and conditioning on the POBS of a CSBS (Total Fill BC Sealer^®^ FKG, Swiss Endo) compared to the one of an epoxy resin-based sealer (AH Plus Jet^®^ Dentsply Sirona).

The first null hypothesis is that there are no differences between the POBS of CSBS and AH Plus Jet^®^. The second null hypothesis is that the irrigation protocol does not impact the POBS values and the failure mode.

## 2. Methods

### 2.1. Sample Size Calculation

The primary outcome variable of the study is the push-out bond strength on root canals. The appropriate Type I error was set at 5.0% and the power was set at 95%. Using the data of a previous study [27], a sample size of 14 in each subgroup was used to detect a significant difference with an estimated effect size of 1.451.

### 2.2. Teeth Selection

This study was approved by the Ethics committee (2019-241). From a pool of 500 extracted premolars for periodontal reasons, 84 human mandibular premolars were included.

Inclusion criteria were defined after careful observation and cone beam-computed tomography (CBCT imaging, Dentsply Sirona, Charlotte, NC, USA) scanning of the teeth; only well-developed single-rooted teeth with Vertucci type I configuration with straight root canal (curvature < 5°) without any previous root canal preparation or obturation and with similar canal size and cross-sectional canal shape were selected. Teeth with cracks, resorptions, caries or previous root canal treatment were excluded from the study.

Samples were then stored in distilled water containing 0.5% thymol until the start of the experiments.

### 2.3. Root Canals Preparation

All treatment procedures were carried out by the same operator experienced in endodontics.

Apical patency was established using a size 10 K-file (VDW, Munich, Germany) and working length (WL) was obtained by gently withdrawing the instrument until reaching the major apical foramen minus 0.5 mm. Then, the crown of each premolar was cut in order to standardize a WL of 17 mm. All root canals were shaped with R25^®^ (Reciproc, VDW, Munich, Germany) according to the manufacturer’s instructions. The instruments were used in a reciprocating motion with an amplitude < 3 mm; the flutes of the instrument were cleaned after three in-and-out movements and the root canal irrigated with 2.5 mL of the selected irrigant and apical patency was rechecked. This sequence was recapitulated until carrying R25^®^ to WL.

### 2.4. Irrigation Protocol

As an irrigation protocol, teeth were divided in three groups:

Group A (n = 28)

During root canal shaping, conventional syringe irrigation was used with 10 mL of 2.5% NaOCl. Micro aspiration inside the root canal and 1 paper point were used to remove NaOCl remnants before the use of 17% EDTA.

Final irrigation was performed using 3 mL of 17% EDTA agitated with a sonic activation device, Endoactivator (Dentsply Maillefer) for 1 min followed by microaspiration and 1 paper point to remove EDTA remnants. Then, the canal was redisinfected using 2.5% NaOCl agitated with Endoactivator for 1 min too. This was followed by a final rinse with 3 mL of 2.5% NaOCl.

Group B (n = 28)

During root canal shaping, the conventional syringe irrigation was used with 10 mL of Dual rinse HEDP, implying prior mixing of HEDP with 2.5% NaOCl, as recommended by the manufacturer.

Final irrigation was performed using the same solution agitated with Endoactivator (Dentsply Maillefer) for 1 min. This was followed by a final rinse with 3 mL of HEDP with 2.5% NaOCl.

Group C (n = 28)

During root canal shaping, conventional syringe irrigation was used with 10 mL of 2.5% NaOCl. Final irrigation was performed using 2.5% NaOCl agitated with Endoactivator (Dentsply Maillefer) for 1 min followed by a final rinse with 3 mL of the same irrigant.

Following irrigation, each root was vertically embedded into acrylic resin before obturation to simulate the periodontal tissues (Technovit 4071, HeraeusKulzer, Hanau, Germany).

The deepest penetration of the conventional needle tip (27 G; 25 mm) was 3 mm short of WL for all the irrigation protocols. After immersion for 1 min in 2.5% NaOCl, matched gutta-percha R25 cones (VDW, Munich, Germany) were fitted and root canals were then dried with paper points at the end of each protocol.

### 2.5. Root Canal Filling

For this step, each group A, B and C were divided into 2 sub-groups (n = 14) according to the type of sealer used (AH Plus Jet^®^ or Total Fill BC Sealer^®^) with the single-cone technique using the injection tip of each sealer, respectively, as follows:

A1: 14 root canals filled with single-cone/AH Plus Jet^®^.
A2: 14 root canals filled with single-cone/Total Fill BC Sealer^®^.
B1: 14 root canals filled with single-cone/AH Plus Jet^®^.
B2: 14 root canals filled with single-cone/Total Fill BC Sealer^®^.
C1: 14 root canals filled with single-cone/AH Plus Jet^®^.
C2: 14 root canals filled with single-cone/Total Fill BC Sealer^®^.

Before injection of Total Fill BC Sealer^®^ in the corresponding groups A2, B2 and C2, the root canals were kept slightly wet (no desiccation of the dentin walls), because CSBSs, unlike conventional sealers, need moisture to initiate their hydration reaction, which conditions their setting process [9].

Each sealer was injected accordingly, in the middle third of every root canal using its specific manufactured tip, before slowly bringing the R25 gutta-percha cone to WL. The gutta-percha cone was then sectioned at the level of the coronal orifice and condensed with a vertical plugger. The sealer was thus completely covered and protected by the gutta-percha.

The access cavities were filled with Cavit G (3M ESPE, Seefeld, Germany) and the teeth were stored in an incubator at 37 °C and 100% humidity for 7 days [28].

**Figure 1:**
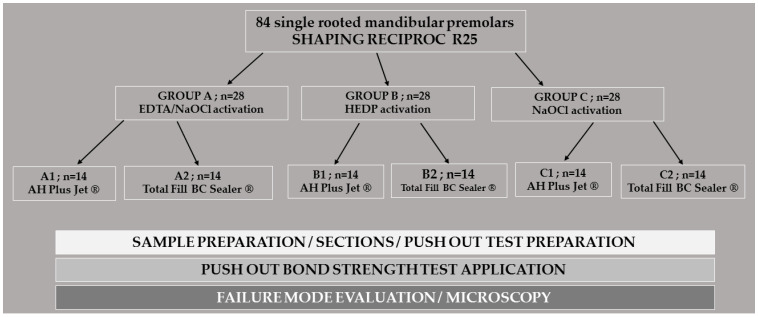
Schematic drawing of flow of the methodology performed for the present study.

The flow of the treatment performed is shown in a schematic drawing (Figure 1).

### 2.6. Push-Out Test Preparation

The roots were sectioned horizontally with a 0.25 mm-low-speed saw (Leitz, Wetzlar, Germany) under permanent water-cooling between a distance of 7.00 mm and 11.75 mm from the apex. Slices of 1 mm thickness were obtained by using a digital caliper from the coronal, middle and apical thirds of the canal.

Only one slice was selected randomly in each third of every root canal and viewed under microscopy with 4× magnification (Olympus BX60, Tokyo, Japan). When a slice revealed multiple wide gaps/voids between gutta-percha and dentinal walls the section was discarded and replaced by another one of the same third in the same root canal, observed under same conditions of microscopy.

### 2.7. Push-Out Bond Strength Application

The specimens were placed in a metallic jig with a hole underneath to allow the canal filling material to fall from the canal after dislodgement.

The vertical load was applied in an apical to coronal direction and generated by a universal testing machine (YLE, GmbH, Frankfurt, Germany) at a speed of 1 mm per minute. A standardized size plunger with a tip diameter of 0.3 mm for the apical third and 0.5 mm for the middle and coronal thirds was used to apply the vertical load on the filling material, with an equal distribution of the load on 60 to 85% of the area [4].

The applied load generated by a software (YLtestS Testing Software) and the bond failure illustrated by a sudden reduction of load, recorded in Newton (N) is shown by a graphic illustration. The POBS of each specimen calculated was expressed in N/mm^2^ (equivalent to MPa).

### 2.8. Failure Mode Evaluation

After dislodgement of the root canal filling, each specimen was observed under 20× magnification using microscopy (Olympus BX60 Japan) and photographs of each specimen were taken afterwards. The photographs were separately evaluated by two calibrated blinded operators and the mode of failure was recorded. In case of disagreement a joint meeting of all authors was made until a consensus was reached.

**Figure 2:**
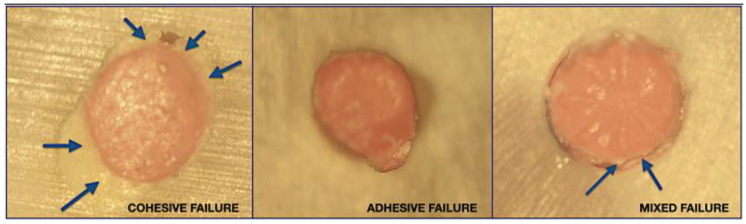
Samples under magnification ×20 as observed just after push-out test illustrating the three possible bond failures. The push out test stops automatically after decohesion of the gutta-percha cone. The latter is nevertheless still visible on the pictures due to its incomplete dislodgment. The blue arrows indicate some areas showing the sealer.

There are three possible categories of failure (Figure 2): adhesive failure (no sealer left on canal walls), cohesive failure (sealer present on entire canal walls) and mixed failure (sealer in patches on canal wall).

## 3. Statistical Analysis

Data analyses were carried out using IBM SPSS Statistics for Windows, version 26 (IBM Corp., Armonk, NY, USA). The level of significance was set at 5%. Descriptive statistics for quantitative and qualitative variables were presented as mean ± standard deviation (SD) and frequency (percentage), respectively. Welch’s ANOVA was used to compare push-out bond strength means among sub-groups or root levels, followed by Games–Howell post-hoc multiple comparison tests. Shapiro–Wilk test was used to assess the normality of distribution of the push-out bond strength variable. Kruskal–Wallis test was used as well, followed by Bonferroni post-hoc test when comparing means within each category (sub-groups or root levels). Chi-square tests were used to evaluate the association between the failure modes and the sub-groups or root levels.

## 4. Results

### 4.1. Influence of the Irrigation Protocol

Table 1 displays the mean POBS for each sub-group regardless of the root level; the highest value was observed for the A2 sub-group, and the lowest for the B2 sub-group. The sub-group A2 showed a push-out bond strength statistically significantly greater than values of sub-groups B2 and C1 (*p* < 0.05), and statistically similar to those of the other sub-groups (A1, B1 and C2). On the other hand, the sub-group B2 showed a push-out bond strength statistically significantly lower than those of sub-groups A2, B1 and C2.

### 4.2. Root Canal Level and Push out Bond Strength

Results of the comparison of POBS means between root levels regardless of the sub-groups are shown in Table 2. The highest mean was observed at the apical level and the lowest at the coronal level. The means were statistically significantly different among root levels (*p* < 0.05).

### 4.3. Impact of Irrigation Protocol Joined with Root Canal Level on POBS

Table 3 displays POBS values according to sub-groups and root levels. The POBS at the apical level was significantly greater than that at the coronal level in every sub-group (*p* < 0.001). No statistically significant differences were found between values of the six sub-groups at the middle level; however, at the coronal level, the sub-group A2 showed a POBS statistically significantly greater than that of the sub-group B2 (*p* < 0.05), and statistically similar to those of the other sub-groups (A1, B1, C1, and C2) (*p* > 0.05). At the apical level, sub-groups A2, B1 and C2 showed statistically significantly greater POBS means than those of sub-groups A1, B2 and C1 (*p* < 0.05). No statistically significant differences were found between values of sub-groups A2, B1 and C2 at the apical level (*p* > 0.05).

### 4.4. Failure Mode

**Figure 3:**
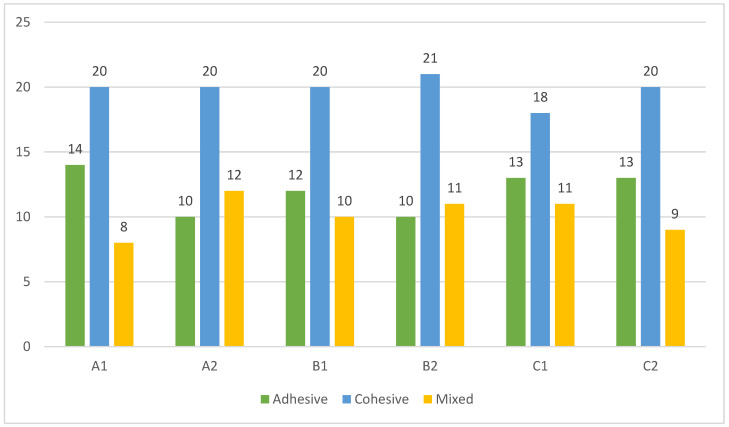
Frequency distribution of the 252 specimens according to the sub-groups and modes of failure.

The frequency distribution of the specimens according to the modes of failure are illustrated in Figure 3; the most common failure mode was the cohesive (47.2%), followed by the adhesive (28.6%) and the mixed (24.2%) mode. There were no statistically significant associations between the mode of failure and the sub-groups (*p* > 0.05); no statistically significant relationship was found between the failure modes and the root levels (*p* > 0.05).

## 5. Discussion

The purpose of the present study was to assess the impact of the irrigation protocol on the POBS of a CSBS, Total Fill BC Sealer^®^ in comparison with the one of AH Plus Jet^®^.

In the present study, the null hypotheses were rejected. Based on our results, EDTA/Total Fill BC Sealer^®^ and NaOCl/Total Fill BC Sealer^®^ showed the best results for POBS of CSBS with a statistical difference when compared with HEDP/Total Fill BC Sealer^®^. This means that irrigation protocol can impact the POBS of this CSBS. Moreover, POBS was significantly higher in the apical third than in the coronal one. The POBS means were statistically significantly different among root levels (*p* < 0.05).

Investigating the POBS is commonly used to evaluate adhesion of endodontic sealers to dentinal walls even if many other protocols have been suggested for this purpose [29,30]. It is well admitted that filling materials must be well adapted to the dentin walls to avoid bacterial leakage [31], leading us to consider that the bond strength of sealers might impact the success of the endodontic therapy [29].

A previous study showed that the single-cone technique might have inherent limitation especially in oval canals regardless of the sealer used [32]. Taking this into consideration, oval shaped root canals of mandibular premolars were selected for their anatomy especially in the middle and coronal thirds [33] in order to simulate the worst clinical scenario.

For proper standardization of the POBS methodology, the ratio between the plunger’s diameter and the specimen’s diameter is recommended to be set between 0.6 and 0.85 [4], which was respected in the present study.

Hydrophilic materials such as CSBS use the smear layer’s moisture as a coupling agent to create hydroxyapatite-like precipitation while setting and chemically bonding to dentin [34].. This may explain why EDTA/NaOCl final irrigation did not enhance POBS of Total Fill BC Sealer^®^ in the present study compared to the use of NaOCl alone. Also, their hydrophilic, hygroscopic nature and their ability to create calcium phosphate deposition may impact the adhesion of CSBS to the dentin walls.

In the present study, the use of EDTA/NaOCl for final irrigation followed by obturation with Total Fill BC Sealer^®^ showed comparable POBS values obtained with EDTA/AH Plus Jet^®^. This means that the use of EDTA resulted in good all-around performance in terms of bond strength for both AH Plus Jet^®^ as well as Total Fill BC Sealer^®^. Similar conclusions were obtained from Carvalho et al. (2017) and Tuncel et al. (2015), who found that the use of different chelating agents for the smear layer removal did not improve the bond strength of AH Plus^®^ compared with iRoot SP^®^, Total Fill BC Sealer^®^ and MTA Fillapex^®^ [16,18]. In fact, increasing the surface roughness for both AH Plus Jet^®^ and Total Fill BC Sealer^®^ using EDTA could be clinically beneficial because this retention is provided by the micromechanical interactions of the endodontic sealer with dentin tubules, leading to their higher dislocation resistance.

As mentioned previously, HEDP also known as etidronic acid, (1-hydroxyethylidene-1,1-diphosphonic acid) is a “soft” chelator that can be used in direct combination with NaOCl to form an all-in-one disinfecting, deproteinizing and chelating irrigant. It has been demonstrated that the continuous chelation irrigation protocol optimizes the bond strength of the epoxy resin-based sealer to dentin [35].

Reduction in smear layer content and debris removal by the mild chelating action of HEDP increased the covalent bond formation with AH Plus^®^ to dentinal walls by exposing the amino group of dentinal collagen [22]. As a matter of fact, the present study proved that HEDP/AH Plus Jet^®^ group resulted in significantly higher POBS when compared with HEDP/TotalFill BC Sealer^®^ that showed the lowest POBS values.

No statistically significant difference was noted between EDTA/AH Plus Jet^®^ and HEDP/AH Plus Jet^®^, with a good performance of the resin-based sealer in terms of adhesion to dental walls. This could be accredited to the continuous chelating action of HEDP enabling the dissolution of organic components of dentin, as well as conditioning of the inorganic part, that led to the same POBS results for AH Plus Jet^®^ when used after EDTA and HEDP. This also explains why there was a statistical significance difference between these results and NaOCl/AH Plus Jet^®^, where no chelation was performed in this group.

Many studies showed that AH Plus^®^ had higher POBS values than CSBS [4,8,16,34]. That is probably because AH Plus^®^ can chemically bond to dentinal collagen amino groups [36,37].

In the present investigation, results of the comparison of POBS means between root levels showed statistically significant differences regardless of the sealers and the irrigation protocol applied. The highest mean was observed at the apical level and the lowest at the coronal level. This should be put into perspective with the anatomy of the root canal, the film thickness of the sealer and the selected root canal filling technique. It is well known that the root canal cross-section in the apical third is relatively round compared with the oval shape of the canal in the coronal third. Likewise, when applying a single-cone technique (with no intra-canal gutta-percha compaction), the film thickness of the sealer is necessarily greater in the coronal and middle third of the root canal than in the apical third where the master cone is well fitted. These considerations may explain the greater POBS values in the apical third regardless of the sealer type and irrigation protocol.

Many studies found that CSBSs were more associated with cohesive failures, which might reflect the evidence of their greater adhesion to dentin walls than gutta-percha [14,15,16]. This is in accordance with the findings of the present study showing that the most frequent failure mode observed was the cohesive failure but with no statistical difference with other failure types.

This study is limited because of its in vitro nature. In fact, sealers were tested after their setting process, while, in reality, the roots are subject to pressure due to masticatory forces directly after obturation. Consequently, POBS study cannot perfectly mimic the clinical conditions [22]. Furthermore, despite ex vivo POBS studies being widely used to evaluate the adhesion of different endodontic sealers to dentin walls and gutta-percha, some limitations have to be highlighted, especially related to the type of gutta-percha used and its roughness and malleability. This may explain the contradictory results when comparing the available studies. Moreover, in terms of clinical relevance, POBS of endodontic sealers should not be considered alone but should be put into perspective with all CSBS physico-chemical and biological properties and related outcomes.

## 6. Conclusions

The activation of EDTA solution resulted in comparable POBS for Total Fill BC Sealer^®^ and AH Plus Jet^®^ whereas the activation of HEDP resulted in the best results for AH Plus Jet^®^.

Within the limitations of this laboratory study, root dentin conditioning using a chelating agent could improve the adhesion quality of AH Plus Jet^®^. According to our results, this might not be the case for Total Fill BC Sealer^®^. Indeed, the activation of EDTA before NaOCl did not improve POBS of Total Fill BC Sealer^®^ compared to the use of NaOCL alone and the use of HEDP for irrigation/activation procedures decreased POBS of this sealer.

Finally, further studies are required to clarify if the use of a chelating agent is mandatory prior to the use of a CSBS. Likewise, long-term clinical outcome investigations are necessary for a better understanding of CSBS behavior in the root canal space.

## Figures and Tables

**Table 1:** Comparison of push-out bond strength means (in MPa) between sub-groups regardless of root levels (n = 252).

Sub-Groups	Push-Out Bond Strength (*Mean ± SD*)	Range (*Minimum*–*Maximum*)	95% CI (*Lower Bound*–*Upper Bound*)	*p*-Value
**A1 (n = 42)**	4.283 ± 3.667 ^ABC^	0.157–14.961	3.140–5.425	<0.001 *
**A2 (n = 42)**	9.631 ± 11.108 ^A^	0.135–41.964	6.170–13.093	
**B1 (n = 42)**	7.102 ± 7.620 ^AB^	0.280–27.537	4.727–9.477	
**B2 (n = 42)**	2.914 ± 2.594 ^C^	0.205–12.600	2.105–3.722	
**C1 (n = 42)**	4.105 ± 3.793 ^BC^	0.335–15.071	2.923–5.287	
**C2 (n = 42)**	7.697 ± 8.137 ^AB^	0.341–33.892	5.162–10.233	

SD = standard deviation; 95% CI = 95% confidence intervals; * *p* < 0.05; different uppercase superscript letters indicate statistically significant differences between values of subgroups.

**Table 2:** Comparison of push-out bond strength means (in MPa) between root levels regardless of the sub-groups (n = 252).

Root Levels	Push-Out Bond Strength (*Mean ± SD*)	Range (*Minimum*–*Maximum*)	95% CI (*Lower Bound*–*Upper Bound*)	*p*-Value
**Coronal** **(n = 84)**	0.980 ± 0.635 ^C^	0.135–2.634	0.842–1.117	<0.001 *
**Middle** **(n = 84)**	3.839 ± 2.063 ^B^	0.829–11.086	3.391–4.286	
**Apical** **(n = 84)**	13.048 ± 8.433 ^A^	1.043–41.964	11.218–14.878	

SD = standard deviation; 95% CI = 95% confidence intervals; * *p* < 0.05; different uppercase superscript letters indicate statistically significant differences between values of root levels.

**Table 3:** Comparisons of push-out bond strength means (in MPa) according to root levels and sub-groups.

	Root Levels	Coronal Level	Middle Level	Apical Level	*p*-Value
Sub-Groups					
**A1: EDTA/AH Plus Jet**		0.938 ± 0.583 ^ABb^	4.012 ± 2.136 ^a^	7.897 ± 3.362 ^Ba^	<0.001 *
**A2: EDTA/Total Fill BC Sealer**		1.361 ± 0.672 ^Ac^	4.718 ± 2.692 ^b^	22.815 ± 9.726 ^Aa^	<0.001 *
**B1: HEDP/AH Plus Jet**		1.318 ± 0.824 ^ABb^	3.346 ± 2.102 ^b^	16.642 ± 5.367 ^Aa^	<0.001 *
**B2: HEDP/Total Fill BC Sealer**		0.588 ± 0.244 ^Bb^	2.805 ± 0.925 ^a^	5.349 ± 2.842 ^Ba^	<0.001 *
**C1: NaOCl/AH Plus Jet**		0.761 ± 0.442 ^ABb^	3.503 ± 1.461 ^a^	8.051 ± 3.722 ^Ba^	<0.001 *
**C2: NaOCl/Total Fill BC Sealer**		0.911 ± 0.589 ^ABc^	4.649 ± 2.202 ^b^	17.533 ± 6.314 ^Aa^	<0.001 *
***p*** **-value**		0.009 *	0.165	<0.001 *	

* *p* < 0.05; different uppercase superscript letters indicate statistically significant differences between values of sub-groups in each root level; different lowercase superscript letters indicate statistically significant differences between values of root levels in each sub-group.

## Data Availability

Not applicable.
